# Supplementary material for: Content-rich biological network constructed by mining PubMed abstracts
Source: BMC Bioinformatics. 2004 Oct 8;5:147. doi: 10.1186/1471-2105-5-147 (PMC528731; doi:10.1186/1471-2105-5-147)
Supplement: Additional File 2 — The original results of the above study (non-essential files are deleted to keep the file size under the limit set by BMC bioinformatics). [file 1471-2105-5-147-S2.bz2 › chilibotAdditionalFile2/dip30/83ID8798570E297/html/EPHB1_EFNB1.html]

 


 **EPHB1** and **EFNB1** 
  
Found 51 abstracts in PubMed, retrieved 30.  
 

 What does Google say? 
 PDF only 
| .edu only 

---

**Interactive relationship** (e.g. stimulation, inhibition, etc)

**Neutral relationship**- Surface densities of ephrin B1  [ **EFNB1** ]  determine  **EphB1**  coupled activation of cell attachment through alphavbeta3 and alpha5beta1 integrins.  Ref: 10205170 EMBO J, 1999
- These results suggest that ephrin B  [ **EFNB1** ]  EphB  [ **EPHB1** ]  interactions play a key role in the sorting of axons at the vertebrate chiasm.  Ref: 10774728 Neuron, 2000
- **EphB1**  transfection conferred ephrin B1  [ **EFNB1** ]  responsive activation of alpha5beta1 integrin mediated cell attachment in human embryonic kidney cells.  Ref: 10205170 EMBO J, 1999
- EphB  [ **EPHB1** ]  receptors influence growth of ephrin B1  [ **EFNB1** ]  positive statoacoustic nerve fibers.  Ref: 12405963 Eur J Neurosci, 2002
- **EphB1**  Fc induced endothelial ephrin B1  [ **EFNB1** ]  tyrosine phosphorylation, migration and integrin mediated alpha v beta 3 and alpha 5 beta 1 attachment and promoted neovascularization, in vivo, in a mouse corneal micropocket assay.  Ref: 12118063 J Cell Sci, 2002
- Recently, we found that NB cell lines expressed several EPHB  [ **EPHB1** ]  and EFNB  [ **EFNB1** ]  transcripts, which encode EPHB  [ **EPHB1** ]  subgroup receptors and ephrin B  [ **EFNB1** ]  subgroup ligands, respectively.  Ref: 10389937 Clin Cancer Res, 1999
- Interactions between these molecules are promiscuous, but they largely fall into two groups EphA receptors bind to GPI anchored ephrin A ligands, while EphB  [ **EPHB1** ]  receptors bind to ephrin B  [ **EFNB1** ]  proteins that have a transmembrane and cytoplasmic domain.  Ref: 10730216 Int Rev Cytol, 2000
- When expressed prematurely in the embryonic chiasm, ephrin B  [ **EFNB1** ]  causes precocious ipsilateral projections from the EphB  [ **EPHB1** ]  expressing RGCs.  Ref: 10774728 Neuron, 2000
- Interactions between these molecules are promiscuous, but largely fall into two groups EphA receptors bind to glycosylphosphatidyl inositol anchored ephrin A ligands, and EphB  [ **EPHB1** ]  receptors bind to transmembrane ephrin B  [ **EFNB1** ]  proteins.  Ref: 11128993 Philos Trans R Soc Lond B Biol Sci, 2000
- Ephrin B1  [ **EFNB1** ]  is expressed by both granule and Purkinje neurons whereas EphB  [ **EPHB1** ]  is present in granule neurons in early postnatal cerebellum at a time coincident with axonal and dendrite outgrowth.  Ref: 12139920 Mol Cell Neurosci, 2002
- Our data suggest that branches of EphB  [ **EPHB1** ]  expressing axons are attracted medially by ephrin B1  [ **EFNB1** ] , and provide molecular mechanisms for D V mapping in visual centers.  Ref: 12165470 Neuron, 2002
- Here,  **EphB1**  regulated cell attachment by discriminating the density at which ephrin B1  [ **EFNB1** ]  was displayed on a reconstituted surface.  Ref: 10205170 EMBO J, 1999
- Ephrin B  [ **EFNB1** ]  proteins have conserved cytoplasmic tyrosine residues that are phosphorylated upon interaction with an EphB  [ **EPHB1** ]  receptor, and may transduce signals that regulate a cellular response.  Ref: 10403252 Nature, 1999
- Ephrin B  [ **EFNB1** ]  EphB  [ **EPHB1** ]  family proteins are implicated in bidirectional signaling and were initially defined through the function of their ectodomain sequences in activating EphB  [ **EPHB1** ]  receptor tyrosine kinases.  Ref: 12118063 J Cell Sci, 2002
- Alternatively, EphB  [ **EPHB1** ]  receptor signaling may act as a ligand density sensor and titrate signaling pathways that promote branch extension toward an optimal ephrin B1  [ **EFNB1** ]  concentration found at the TZ.  Ref: 12702655 Development, 2003
- Stably transfected 3T3 cells overexpressing ephrin B1  [ **EFNB1** ]  enhance survival and neurite growth from cultured cerebellar granule neurons, an effect that is inhibited by the presence of an excess of a soluble EphB  [ **EPHB1** ]  protein.  Ref: 12139920 Mol Cell Neurosci, 2002

**Non-interactive relationship** (e.g. studied together, co-existance, homology, etc.)

- METHODS Reverse transcriptase polymerase chain reaction RT PCR, Northern Blot analyses, and immunohistochemistry were used to examine 11 colon carcinoma cell lines and 20 human colon carcinoma specimens with adjacent uninvolved mucosa for the expression of EphB  [ **EPHB1** ]  and ephrin B  [ **EFNB1** ]  family members.  Ref: 11920461 Cancer, 2002
- The distributions of the Eph class receptors EphA4 and  **EphB1** , and their ligands ephrin A2, ephrin B1  [ **EFNB1** ] , and ephrin B2, were analysed by immunostaining in the mouse inner ear.  Ref: 12115675 J Comp Neurol, 2002
- **EphB1**  and its potential ligands ephrin B1  [ **EFNB1** ]  and ephrin B2 showed a segregated layered expression in the lateral wall of the cochlear duct the external sulcus, which together with EphA4 expressed in the area, form a four layered structure with an alternating pattern of receptors and ligands in the different layers.  Ref: 12115675 J Comp Neurol, 2002
- EphB  [ **EPHB1** ]  receptors are expressed in a low to high DV gradient by retinal ganglion cells RGCs, and ephrin B1  [ **EFNB1** ]  is expressed in a low to high LM gradient in the tectum.  Ref: 12702655 Development, 2003
- We propose that Xdsh, which is expressed in various tissues, is involved in EphB  [ **EPHB1** ]  and ephrin B  [ **EFNB1** ]  signaling related to regulation of cell repulsion via modification of RhoA activity.  Ref: 12574121 EMBO J, 2003
- Activation competent but signaling defective  **EphB1**  point mutants failed to stimulate ephrin B1  [ **EFNB1** ]  dependent attachment.  Ref: 10205170 EMBO J, 1999
- In contrast, stimulation of Jurkat cells with the EphB  [ **EPHB1** ]  receptor ligand ephrin B1  [ **EFNB1** ]  does not cause Cbl phosphorylation.  Ref: 12794130 J Immunol, 2003
- We found that several EPHB  [ **EPHB1** ]  and EFNB  [ **EFNB1** ]  transcripts were expressed together in all NBs examined.  Ref: 11464911 Med Pediatr Oncol, 2001
- Here we use a soluble  **EphB1**  ectodomain fusion protein  **EphB1**  Fc to demonstrate that ephrin B1  [ **EFNB1** ]  transduces signals that regulate cell attachment and migration.  Ref: 12118063 J Cell Sci, 2002
- In vivo disruption of EphB  [ **EPHB1** ]  ephrin B  [ **EFNB1** ]  function by application of exogenous EphB  [ **EPHB1** ]  or expression of dominant negative ephrin B  [ **EFNB1** ]  ligand in dorsal retinal axons causes these axons to shift dorsally in the tectum, while misexpression of wild type ephrin B  [ **EFNB1** ]  in ventral axons causes them to shift ventrally.  Ref: 12165469 Neuron, 2002
- Activation of ephrin B1  [ **EFNB1** ]  by  **EphB1**  Fc induced phosphorylation of p46 JNK but not ERK 1 2 or p38 MAPkinases.  Ref: 12118063 J Cell Sci, 2002
- in this issue of Neuron provide evidence for both forward and reverse ephrin B  [ **EFNB1** ]  EphB  [ **EPHB1** ]  signaling in regulating D V topography.  Ref: 12165462 Neuron, 2002
- In adhesion assays, engagement of ephrin B  [ **EFNB1** ]  ligands to EphB  [ **EPHB1** ]  receptors increases melanoblast attachment to fibronectin.  Ref: 12117812 Development, 2002
- PROCEDURE In this study, we examined the expression of EPHB  [ **EPHB1** ]  and EFNB  [ **EFNB1** ]  transcripts in 24 NB specimens representing all clinical stages.  Ref: 11464911 Med Pediatr Oncol, 2001
- A major recent advance is the demonstration that EphB  [ **EPHB1** ]  receptor forward signaling and ephrin B  [ **EFNB1** ]  reverse signaling mediate axon attraction to control dorsal ventral retinal mapping along the lateral medial tectal axis.  Ref: 12593983 Curr Opin Neurobiol, 2003
- We recently found that transcripts encoding the EPHB  [ **EPHB1** ]  subgroup EPHB  [ **EPHB1** ]  and the ephrin B  [ **EFNB1** ]  subgroup EFNB  [ **EFNB1** ]  were expressed together in neuroblastoma NB cell lines.  Ref: 11464911 Med Pediatr Oncol, 2001
- Ephrin B  [ **EFNB1** ]  and EphB  [ **EPHB1** ]  are distributed in matching dorsoventral gradients in the embryonic Xenopus visual system with retinal axons bearing high levels of ligand dorsal projecting to tectal regions with high receptor expression ventral.  Ref: 12165469 Neuron, 2002
- Using reverse transcription polymerase chain reaction RT PCR, Western blot and immunocytochemical analyses, we identified distinct and reciprocal expression patterns of full length isoforms of EphA5, EphA6, EphA7,  **EphB1** , ephrin A2 and ephrin B1  [ **EFNB1** ]  that correlated with structural features of the peripheral vestibular system in adult rats.  Ref: 10792438 Eur J Neurosci, 2000
- **EphB1**  ephrin B1  [ **EFNB1** ]  engagement did not promote cell attachment through mechanical tethering, but did activate integrin mediated attachment.  Ref: 10205170 EMBO J, 1999
- We report that the EphB  [ **EPHB1** ]  receptor ligand, ephrin B1  [ **EFNB1** ] , may act bifunctionally as both a branch repellent and attractant to control the unique mechanisms in mapping the dorsal ventral DV retinal axis along the lateral medial LM axis of the optic tectum.  Ref: 12702655 Development, 2003
- To explore the role of EPHB  [ **EPHB1** ]  receptors and ephrin B  [ **EFNB1** ]  ligands in the biology of NB, we examined the expression of EPHB  [ **EPHB1** ]  and EFNB  [ **EFNB1** ]  transcripts in 47 primary NB specimens.  Ref: 10389937 Clin Cancer Res, 1999
- Together, these results suggest a role for EphB  [ **EPHB1** ]  receptors in providing guidance signals to ephrin B1  [ **EFNB1** ]  positive SAG nerve fibers.  Ref: 12405963 Eur J Neurosci, 2002
- We conclude that in the intestinal epithelium beta catenin and TCF couple proliferation and differentiation to the sorting of cell populations through the EphB  [ **EPHB1** ]  ephrin B  [ **EFNB1** ]  system.  Ref: 12408869 Cell, 2002
- Multiple EPHB  [ **EPHB1** ]  and EFNB  [ **EFNB1** ]  transcripts were expressed in all of the NB tumors examined, suggesting the involvement of these transcripts in modulating the biological behavior of NB.  Ref: 10389937 Clin Cancer Res, 1999
- The expression patterns of transmembrane ephrin B  [ **EFNB1** ]  ligands and interacting EphB  [ **EPHB1** ]  receptors suggest that these proteins are likely candidates for coordinating the segmentation of spinal motor axons and neural crest cells.  Ref: 10699981 J Neurobiol, 2000
